# Differences in clinical characteristics and prognosis of Penicilliosis among HIV-negative patients with or without underlying disease in Southern China: a retrospective study

**DOI:** 10.1186/s12879-015-1243-y

**Published:** 2015-11-16

**Authors:** Ye Qiu, Haifei Liao, Jianquan Zhang, Xiaoning Zhong, Caimei Tan, Decheng Lu

**Affiliations:** Department of Integrated Medicine, The Affiliated Tumor Hospital of Guangxi Medical University, Nanning, Guangxi China; Department of Respiratory Medicine, The First Affiliated Hospital of Guangxi Medical University, Nanning, Guangxi 530021 China; Department of Endocrinology Medicine, The First Affiliated Hospital of Guangxi Medical University, Nanning, Guangxi China

**Keywords:** *Penicillium marneffei*, HIV-negative, Underlying diseases, CD4 T-cellular immunodeficiency, Prognosis

## Abstract

**Background:**

The incidence of Penicillium marneffei infection has recently increased. This fungus can cause fatal systemic mycosis in both immunocompetent and immunocompromised patients without HIV infection.

**Methods:**

We retrospectively analysed *Penicilliosis* patients between January 1, 2003 and August 1, 2014 at the First Affiliated Hospital of Guangxi Medical University. HIV-negative patients with *Penicilliosis* were divided into two groups: patients with underlying disease (Group D) and patients without underlying disease (Group ND). HIV-positive patients were excluded. The relationships between overall survival and the study variables were assessed using univariate and multivariate analyses.

**Results:**

During 11 years, *Penicillium marneffei* infection was diagnosed in 109 patients. Sixty-six (60.55 %) patients were HIV-positive and excluded from these cases. Forty-three patients were HIV-negative were enrolled. Among these patients, 18 (41.86 %) patients were in Group D, and 25 (58.14 %) were in Group ND. The most common underlying disease was diabetes. There were no statistically significant differences between the two groups in clinical characteristics, except for immune state and prognosis. Group ND had higher lymphocyte cell counts, CD4 cell counts, and CD4 T-cell percentages than Group D (P < 0.05). Patients in Group D had higher recurrence and mortality rates than Group ND (P < 0.05). In the univariate analysis, only underlying disease, CD4 cell percentage, and T lymphocyte cell percentage were significantly associated with overall survival.

**Conclusions:**

*Penicillium m*arneffei can infect HIV-negative patients and can cause fatal systemic mycosis. There were no clear differences in clinical manifestations among HIV-negative patients with and without underlying disease. However, *Penicillium marneffei* in HIV-negative patients in with underlying diseases may cause immune function decline and a deficiency in T-cell-mediated immunity. Underlying disease, CD4 cell percentage, and T lymphocyte cell percentage may be potential risk factors affecting prognosis. Timely, effective, and longer courses of antifungal treatments are important in improving prognoses.

## Background

*Penicillium marneffei* is capable of causing fatal systemic mycosis in immunocompromised individuals, especially in HIV-positive patients [1–3]. However, the incidence of *Penicillium marneffei* infection in both immunocompetent and immunocompromised patients without HIV infection has shown a marked increase in recent years [3]. *Penicillium marneffei* infection can cause immune function decline and deficiency in T-cell-mediated immunity even in a healthy host, such as haematological malignancies, colon cancer, myasthenia gravis, mixed connective tissue disease, transplant rejection, systemic lupus erythematosus, diabetes mellitus, and corticosteroids or immunosuppressive agents [3, 4]. There are numerous retrospective studies investigating the differences of penicilliosis among patients with and without HIV infection [3–8]. However, no studies have observed differences in *Penicillium marneffei* infection among HIV-negative patients with and without underlying disease. This retrospective study is the first time to describe differences in clinical features, immune status, treatment, and outcomes and to elucidate the important factors that influence successful treatment and prognosis of HIV-negative patients with penicilliosis with and without underlying disease.

## Methods

### Patient population

A retrospective study was conducted between January 1, 2003 and August 1, 2014 at the First Affiliated Hospital of Guangxi Medical University. Consecutive patients diagnosed with *Penicilliosis* were eligible. HIV-negative patients with *Penicillium marneffei* infection were included and divided into two groups: patients with underlying disease (Group D) and those without underlying disease (Group ND). Patients who were HIV-positive were excluded. The patients’ clinical records were reviewed for basic information, medical history, auxiliary examination results, and treatments, and summarised for analysis.

This study was approved by the Faculty of Medicine, The First Affiliated Hospital of Guangxi Medical University Ethical Committee. All patients provided written informed consent.

### Diagnosis criteria for *Penicillium marneffei* infection

There were two methods used for pathological and pathogen examination. In the first method, cultures of clinical specimens, including blood, sputum, lymph node, lung tissue, bone, and bone marrow, were established on Sabouraud’s dextrose agar at 25 °C and 37 °C. Positive cultures for *Penicillium marneffei* were characterised by dimorphic fungi that grew as a mould at 25 °C and as yeast at 37 °C. A unique characteristic of *Penicillium marneffei* mould is the presence of a soluble red pigment that diffuses into the agar making the reverse side appear either pink or red at 25 °C [2].

For the second method, the yeast form of *Penicillium marneffei* was identified by cytology and histopathology from tissues and secretions by periodic acid-Schiff staining or Wright’s staining. *Penicillium marneffei* has a characteristic morphology, including a transverse septum [6].

Patients’ sera were tested in duplicate at our hospital and the Guangxi Center for Prevention and Control by using enzyme-linked immunosorbent assay (Enzymun-Test Anti-HIV 1 + 2; Boehringer Mannheim GmbH Diagnostica) and particle agglutination test (Serodia-HIV; Fujirebio Inc., Tokyo, Japan).

### Inclusion and exclusion criteria

Inclusion criteria were as follows: i) HIV negative; and ii) a clear diagnosis of *Penicillium marneffei* infection.

Patients diagnosed with *Penicillium marneffei* infection but who were HIV-positive were excluded.

### Statistical analysis

Clinical data were analysed as percentages (%), means and standard deviations, and medians and interquartile ranges, as appropriate. Comparison of the demographic data and clinical characteristics of HIV-negative patients with and without underlying diseases was performed using the Student’s *t*-test, Mann–Whitney *U* test, chi-square test, or Fisher’s exact test, as appropriate. Survival curves were estimated using Kaplan-Meier analyses, and the differences in survival rates between the two groups were compared using the log-rank test. Univariate analysis was performed to assess significant differences in clinical characteristics that influence overall survival. Multivariate analysis was performed using Cox regression analysis for significant variables identified by the univariate analysis. All statistical analyses were performed using the Statistical Package for the Social Sciences (Windows version 16.0; SPSS Inc., Chicago, IL, US). Two-tailed tests were used, with a *P*-value of < 0.05 indicating statistical significance.

## Results

### Baseline

During the study period of 11 years, 109 patients were diagnosed with *Penicillium marneffei* infection. Sixty-six (60.55 %) patients were HIV-positive and excluded from this case series. The 43 (39.45 %) remaining patients were HIV-negative and were enrolled in this study. Among these patients, 18 patients (41.86 %) had underlying disease, most commonly diabetes, and were included in Group D; the 25 (58.14 %) remaining patients were in Group ND. Table 1 compares underlying disease and demographic data between the two groups.

**Table 1:** Underlying disease and demographic data of HIV-negative patients with *penicilliosis*

Variables	Group D (*N* = 18)	Group ND (*N* = 25)	*P*-values
Age, years	42.5 (1–68)	38.74y (2–64)	0.348
Male	14 (77.78 %)	16 (64 %)	
Underlying diseases*	18 (100 %)	0	
Diabetes	6 (33.33 %)	0	
Lymphoma	1 (5.56 %)	0	
β-thalassemia	2 (11.11 %)	0	
Breast cancer	1 (5.56 %)	0	
Previous glucocorticoid therapy	1 (5.56 %)	0	
Langerhans cell histiocytosis	1 (5.56 %)	0	
G-6PD deficiency	1 (5.56 %)	0	
Systemic lupus erythematosus	1 (5.56 %)	0	
Subacute thyroiditis	1 (5.56 %)	0	
Chronic hepatitis B	2 (11.11 %)	0	
Hyperthyroidism	2 (11.11 %)	0	

Data are presented as n (%). *One patient may have ≥ 1 underlying diseases

Patients with *Penicillium marneffei* infection were divided into two groups; Group D includes patients with underlying disease, and Group ND includes patients without underlying disease

### Clinical features

**Fig. 1:**
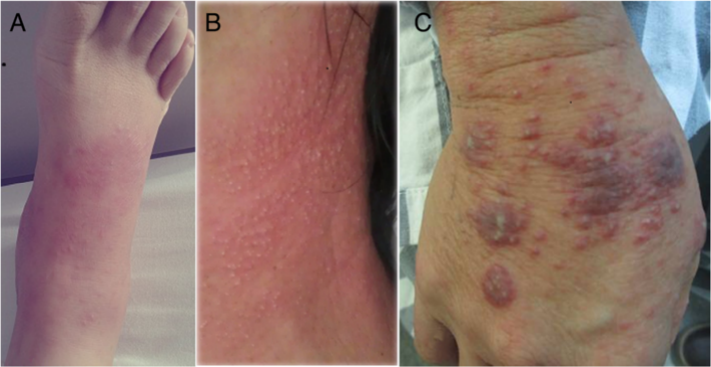
Skin lesions associated with *Penicillium marneffei* infection. Chronic ulcers **a**, pustular psoriasis **b**, and subcutaneous abscesses **c**

Table 2 shows the common clinical manifestations in all HIV-negative patients, including anaemia (40 patients, 93 %), hyperpyrexia (39 patients, 90.6 %), cough (38 patients, 88.3 %), lymphadenopathy (34 patients, 79 %), cutaneous lesions (30 patients, 69.7 %), hepatomegaly (17 patients, 39.5 %), and splenomegaly (16 patients, 37.2 %). The patients in Group ND more commonly exhibited thoracalgia than those in Group D (P < 0.05). Cutaneous lesions consisted of skin rash (Fig. 1a), chronic ulcers, pustular psoriasis (Fig. 1b–c), subcutaneous abscess, panniculitis, and subcutaneous nodules. Symptoms and the the mean time since diagnosis did not differ between the two groups (P > 0.05). However, patients in Group D usually exhibited skin rash distributed over the whole body, while those in Group ND presented with skin ulcers and subcutaneous nodules limited to the face, neck, or limbs.

**Table 2:** Clinical features of *penicilliosis* among HIV-negative patients with and without underlying disease

Variables	Group D (*N* = 18)	Group ND (*N* = 25)	*P*-values
Fever	17 (94.4 %)	22 (88 %)	0.853
Mean temperature	39.58 °C	39.67 °C	0.971
Lymphadenopathy	16 (88.9 %)	18 (72 %)	0.336
Hepatomegaly	10 (55 %)	7 (28 %)	0.068
Splenomegaly	9 (50 %)	7 (28 %)	0.141
Cutaneous lesions	14 (77.8 %)	16 (64 %)	0.332
Cough	15 (83.3 %)	23 (92 %)	0.695
Dyspnoea	6 (33.3 %)	8 (32 %)	0.972
Anaemia	16 (88.9 %)	24 (96 %)	0.767
Osteolysis	8 (44.4 %)	6 (24 %)	0.158
Thoracalgia	8 (44.4 %)	4 (16 %)	0.040*
Bloody stools/diarrhoea	5 (27.7 %)	4 (16 %)	0.578
Duration of diagnosis (days) (median, IQR)	135.5 (27–1008)	102 (12–635)	0.079

Data are presented as n (%) or median (IQR), unless indicated otherwise

*P < 0.05

Patients with *Penicillium marneffei* infection were divided into two groups; Group D included patients with underlying disease, and Group ND included patients without underlying disease

### Laboratory findings

All cases involved anaemia, and 17 patients had decreased platelets (Table 3). The number of white blood cells and neutrophils increased remarkably in both groups and did not differ significantly between groups (P > 0.05). However, Group ND had significantly more lymphocyte cells than Group D (P = 0.023). The CD8 cell counts, CD8 T-cell percentages, and serum immunoglobulin levels did not differ between the two groups (P > 0.05). The mean CD4 cell count was below the normal level in Group D. Furthermore, Group D had fewer lymphocyte cells, CD4 cells, and a lower CD4 T-cell percentage than Group ND (P < 0.05). The most common method of diagnosis was cultures from biopsy tissues and secretions, followed by biopsy pathology, bone marrow cultures, and blood cultures. Positive cultures and histopathology in clinical specimens, including blood, lymph node, bronchoalveolar lavage fluid, lung tissue, bone marrow, bone, and skin, did not differ between groups (P > 0.05). However, purulent secretion cultures had a higher positive rate in Group ND than in Group D (P < 0.05).

**Table 3:** Laboratory findings of *penicilliosis* among HIV-negative patients with and without underlying disease

Variables	Group D (*N* = 18)	Group ND (*N* = 25)	*P*-values
White blood cells (× 10^9^ cells/L)	22.26 (1.89–57.48)	21.56 (5.6–49.7)	0.846
Neutrophils	16.98 (0.67–46.1)	16.53 (3.62–37.87)	0.888
Lymphocyte cells (× 10^9^ cells/L)	1.75	2.88	0.023*
Haemoglobin (g/L)	90.55 ± 21.9	85.75 ± 21.5	0.479
Platelet count (× 10^9^ cells/L)	274.58 (2.6–516)	284 (18–644)	0.493
ALT (U/L)	37.25	35.36	0.979
ALB (g/L)	19.72	23.33	0.325
CD4 T cell count (cells/L)	255.6	623.66	0.000*
CD8 T cell count (cells/L)	389.43	205.00	0.335
CD4%	26.26	34.40	0.004*
CD8%	26.11	23.25	0.394
T cell%	56.51	66.76	0.019*
IgG	25.37	23.58	0.805
Positive cultures	14	22	0.633
-Blood	6/13	6/14	1.000
-Lymph node	0	1/2	
-BAL fluid/lung tissue	0	1/6	
-Bone marrow	2/5	2/5	1.000
-Skin	3/5	4/5	1.000
-Sputum	1/6	1/6	1.000
-Purulent secretions	2/7	7/8	0.041*
Positive histopathology	9	11	0.697
-Lymph node	3/7	3/10	0.644
-Bone marrow/bone	3/7	2/4	1.000
-Skin	3/3	3/10	0.392
-Lung tissue	0/3	2/4	0.286

Patients with *Penicillium marneffei* infection were divided into two groups; Group D included patients with underlying disease, and Group ND included patients without underlying disease

*ALB* serum albumin, *ALT* alanine aminotransferase, *CD*4% CD4 cell percentage, *CD*8% CD8 cell percentage, *T cell*% T lymphocyte cell percentage, *IgG* serum immunoglobulin G. Data are presented as the number of observations or the mean ± standard deviation. *P < 0.05

### Imaging examinations

**Fig. 2:**
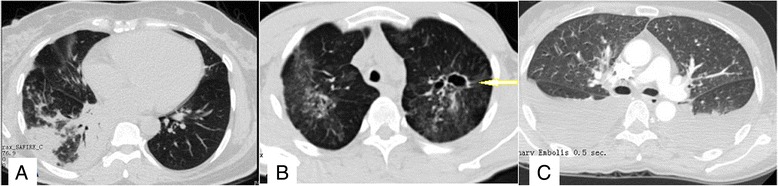
High-resolution computed tomography. High-resolution computed tomography shows interstitial infiltration and alveolar infiltration **a**, a cavitary lesion (arrow) **b**, and pleural effusion **c**

Serous effusion was present in 35 (81.39 %) cases, mainly in the chest or abdominal cavity, though it also occurred in the pericardium and pelvic areas. The effusion was positive for the Rivalta test, with an increase in the number of nucleated cells, suggesting exudate effusion. High-resolution computed tomography (Table 4) showed interstitial infiltration (Fig. 2a); alveolar infiltration was most common, including patchy opacities, fibrous cord proliferation, cavitary lesions (Fig. 2b), mediastinal lymph nodes, and pleural effusion (Fig. 2c).

**Table 4:** High-resolution computed tomography characteristics of *penicilliosis* among HIV-negative patients with and without underlying disease

Variables	Group D (*N* = 18)	Group ND (*N* = 25)	*P*-values
-Normal	4 (22.22 %)	2 (8 %)	0.218
-Interstitial infiltration	9 (50 %)	9 (36 %)	0.359
-Alveolar infiltration	10 (55.56 %)	13 (52 %)	0.818
-Pleural effusion	9 (50 %)	12 (48 %)	0.897
-Cavitary lesion	3 (16.67 %)	1 (4 %)	0.158
-Peritoneal effusion	6 (33.33 %)	8 (32 %)	0.927
Osteolytic bone destruction	8 (44.44 %)	6 (24 %)	0.198

Patients with *Penicillium marneffei* infection were divided into two groups; Group D included patients with underlying disease, and Group ND included patients without underlying disease

Data are presented as *n* (%)

**Fig. 3:**
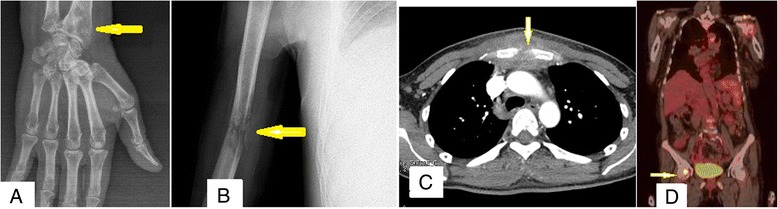
Imaging examinations. Multiple spots or patchy farfetched like bone destruction, periosteal proliferation, fracture, and swelling in surrounding soft tissue was shown *via* radiography **a** & **b**, emission computed tomography, high resolution computed tomography **c**, and positron emission tomography/computed tomography **d**

Osteolytic bone destruction in 14 (32.55 %) cases (8 patients in Group D and 6 in Group ND) was accompanied by pain and dysfunction. Multiple spots or patchy farfetched bone destruction, periosteal proliferation, fracture, and swollen surrounding soft tissue were present in X-ray images (Fig. 3a–b), emission computed tomography bone scans, high-resolution computed tomography (Fig. 3c), and positron emission tomography/computed tomography (Fig. 3d). After effective antifungal treatment, fractures typically healed within two months, and the patchy lesions were absorbed.

### Antifungal therapy and outcomes

Four patients in Group D and 2 patients in Group ND ceased antifungal treatment due to serious complications related to *Penicillium marneffei* infection, including multiple organ failure and acute respiratory failure. Among all 43 cases, 37 received antifungal treatment. The majority of patients in this study were treated with intravenous amphotericin B or fluconazole followed by oral itraconazole (Table 5). The mortality rate was 44.44 % (8 patients) and 12 % (3 patients) among those with and without underlying disease, respectively. Recovery occurred in 33.33 % (6 patients) and 28 % (7 patients) of patients with and without underlying disease, respectively. The longest duration before relapse was 4 years. Causes of death for 8 patients in Group D included *Penicillium marneffei* (n = 2), acute respiratory failure (n = 1), and multiple organ failure (n = 5). The cause of death for 3 patients in Group ND was discontinuing treatment because of multiple organ failure. Compared to Group ND patients, Group D patients had higher recurrence and mortality rates (*P* = 0.022).

**Table 5:** Treatment outcomes among HIV-negative patients with and without underlying disease

Variables	Group D (*N* = 18)	Group ND (*N* = 25)	*P*-values
Treatment received	14	23	0.378
Deterioration (discontinue treatment)	4	2	
Medication			
-Amphotericin B then itraconazole	5	6	1.000
-Itraconazole	4	2	0.378
-Fluconazole then itraconazole	4	10	0.220
-Voriconazole	1	5	0.367
Outcome, *n* (%)			0.022*
-Cure	5 (27.78)	13 (52 %)	
-Recovery	6 (33.33 %)	7 (28 %)	
-Dead	8 (44.44 %)	3 (12 %)	

Patients with *Penicillium marneffei* infection were divided into two groups; Group D included patients with underlying disease, and Group ND included patients without underlying disease

*P < 0.05

Data are presented as the number of patients, unless otherwise indicated

### Overall survival

**Fig. 4:**
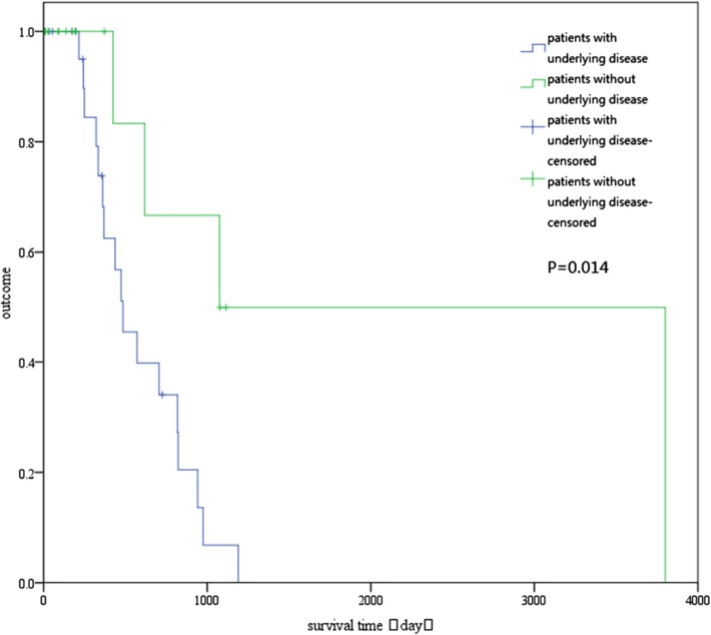
Overall survival of patients with and without underlying disease. The difference between the two groups was statistically significant (log-rank test: *P* = 0.014)

**Fig. 5:**
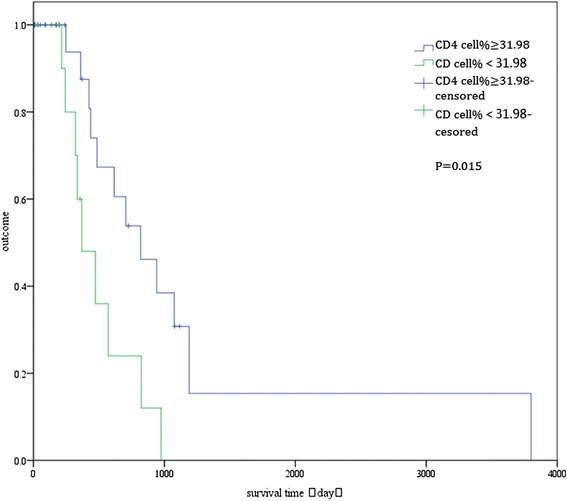
Overall survival of patients with CD4 cell percentage > 31.98 % and ≤ 31.98 %. The difference between the two groups was statistically significant (log-rank test: *P* = 0.015)

**Fig. 6:**
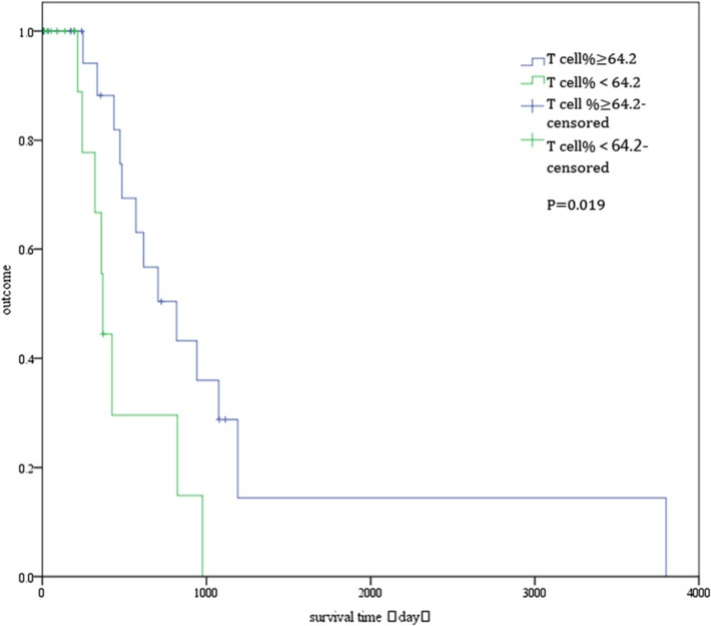
Overall survival curves of patients with T lymphocyte cell percentage (T cell%) > 64.2 % and ≤ 64.2 %. The difference between the two groups was statistically significant (log-rank test: *P* = 0.019)

At the median follow-up of 580.95 days (range, 4–2,345 days), 21 patients (48.83 %) remained alive. To identify predictors of survival, the prognostic values of 27 variables were evaluated (Table 6). In the univariate analysis, only underlying disease, CD4 cell percentage, and T lymphocyte cell percentage were significantly associated with overall survival (*P* < 0.05; Table 1). The long-term survival was significantly better for patients without underlying disease than for patients with underlying disease (log-rank test: *P* = 0.014; Fig. 4). Patients with CD4 cell percentage ≥ 31.98 % had better long-term survival than did patients with CD4 cell percentage < 31.98 % (log-rank test: *P* = 0.015; Fig. 5). Also, patients with T lymphocyte cell percentage ≥ 64.2 % had better long-term survival than did patients with T lymphocyte cell percentage < 64.2 % (log-rank test: *P* = 0.019; Fig. 6). In the multivariate Cox regression analysis, underlying disease, CD4 cell percentage, and T lymphocyte cell percentage were significantly associated with overall survival (*P* > 0.05; Table 6).

**Table 6:** Univariate and multivariate analyses of factors predicting recurrence-free survival of *penicilliosis* among HIV-negative patients

Factor	Univariate			Multivariate		
	HR	95 % CI	*P*	HR	95 % CI	*P*
Age > 60 or < 3 years	0.700	0.274–1.792	0.457			
Underlying diseases	0.228	0.064–0.813	0.014*	0.289	0.074-1.132	0.075
Fever	0.300	0.065–1.378	0.122			
Lymphadenopathy	2.074	0.601–7.155	0.249			
Hepatomegaly	0.374	0.118–1.185	0.095			
Splenomegaly	0.606	0.217–1.697	0.341			
Cutaneous lesions	0.575	0.225–1.471	0.248			
Cough	6.568	0.861–50.100	0.069			
Dyspnoea	1.265	0.479–3.344	0.635			
Anaemia	1.525	0.439–5.303	0.507			
Osteolysis	0.581	0.209–1.618	0.299			
Thoracalgia	1.204	0.453–3.200	0.710			
Bloody stools/diarrhoea	1.122	0.326–3.869	0.855			
Duration of diagnosis	1.026	0.405–2.597	0.957			
WBC > 10*10^9^/L	0.628	0.181–2.177	0.463			
Neutrophils > 6.3*10^9^/L	0.573	0.205–1.604	0.289			
Lymphocytes < 1.10*10^9^/L	2.093	0.730–6.004	0.169			
Haemoglobin < 130 g/L	1.082	0.410–2.855	0.874			
ALT > 45U/L	2.941	0.608–14.230	0.180			
ALB < 55U/L	1.274	0.288–5.627	0.749			
CD4 < 410/ul	1.003	0.998–1.008	0.289			
CD8 < 190 /ul	0.994	0.985–1.002	0.146			
CD4% < 31.98 %	3.033	1.186–7.754	0.015*	1.224	0.305–4.921	0.775
CD8% < 20.70 %	0.654	0.256–1.121	0.374			
T cell% < 64.2 %	2.963	1.146–7.659	0.019*	1.950	0.504–7.539	0.333
IgG < 7 g/L	0.800	0.181–3.536	0.769			
Chest imaging	0.708	0.251–1.999	0.515			

Patients with *Penicillium marneffei* infection were divided into two groups; Group D included patients with underlying disease, and Group ND included patients without underlying disease

*ALB* serum albumin, *ALT* alanine aminotransferase, *CI* confidence interval, *HR* hazard ratio, *WBC* white blood cell, *CD*4 CD4 T cell count, *CD*8 CD8 T cell count, *CD*4% CD4 cell percentage, *CD*8% CD8 cell percentage, *T cell*% T lymphocyte cell percentage, *IgG* serum immunoglobulin G. *P < 0.05

## Discussion

*Penicilliosis* has been reported in immunocompromised patients with HIV infection, but also in patients free of HIV infection. Certain diseases can cause immune function decline and immune disorders, such as haematological malignancies, colon cancer, myasthenia gravis, mixed connective tissue disease, transplant rejection, systemic lupus erythematosus, diabetes mellitus, as well as the use of corticosteroids or immunosuppressive agents, even in HIV-negative [3, 4]. The incidence of *Penicillium marneffei* infection in patients without HIV infection has shown a marked increased in recent years [3, 5–8]. In our study, during an 11-year period, *Penicillium marneffei* infection was present in 43 (43/109, 39.45 %) patients without HIV infection. Among these patients, 41.86 % had underlying diseases, with diabetes being the most common; 58.14 % had no underlying diseases, which suggests that *Penicillium marneffei* infection is not a rare entity, but is also common in HIV-negative individuals and even healthy hosts.

The common clinical manifestations among HIV-negative patients were similar to those in previous reports [6]. However, there were no statistically significant differences between Group D and Group ND in clinical manifestations and laboratory findings, with the exception of thoracalgia, which was more common in HIV-negative patients with underlying disease (*P* < 0.05).

Previous work has postulated that deficiency of CD4+ T-cell-mediated immunity plays a key pathogenic role in AIDS patients and may also be involved in immunocompromised HIV-negative patients, potentially leading to *Penicillium marneffei* infection [6–9]. In our study, CD8 T-cell counts, CD8 T-cell percentages, and serum immunoglobulin levels did not differ between the two groups (*P* > 0.05). However, HIV-negative patients without underlying disease had higher lymphocyte cell counts, CD4 cell counts, and CD4 T-cell percentages than those with underlying disease. The mean CD4 cell count was below normal levels in HIV-negative patients with underlying disease. In addition, in the univariate analysis, only underlying disease, CD4 cell percentage, and T lymphocyte cell percentage were significantly associated with overall survival. Thus, HIV-negative patients with underlying disease, such as cancer, mixed connective tissue disease, transplant rejection, systemic lupus erythematosus, diabetes mellitus, and Langerhans cell histiocytosis, and those taking corticosteroids or immunosuppressive agents, exhibited worse CD4 T-cellular immunodeficiency during the course of the disease than HIV-negative patients without underlying disease. Browne et al. and Lee et al. reported that autoantibody to interferon-ɤ is associated with the new clinical syndrome of adult-onset immunodeficiency [8, 10, 11]. This may be another cause of cell-mediated immunity defects in patients who are not infected with HIV. This also suggests that underlying disease, CD4 cell percentage, and T lymphocyte cell percentage may be potential risk factors affecting prognosis.

Specifically, 32.5 % of patients had osteolytic lesions, and all of these were HIV-negative individuals; no HIV-positive patients exhibited such lesions. Thus, osteolytic lesions are one of the most common and important clinical characteristics of *Penicillium marneffei* infection, which can easily be neglected in HIV-negative patients [12]. Serous effusions are often thought to reflect hypoalbuminaemia, connective tissue disease, or tuberculosis. However, in our study, 81.39 % of patients had multi-exudate effusion, which suggests that exudate effusions are equally common in *Penicillium marneffei* infection in HIV-negative individuals. However, the mechanism by which effusion is generated is unclear; the cause may be inflammation initiated by disseminated *Penicillium marneffei* infection, or may directly involve the thoracic cavity.

The most common diagnostic method of *Penicilliosis* was cultures from biopsy tissues and secretions, followed by biopsy pathology, bone marrow cultures, and blood cultures, which is consistent with the presence of skin lesions and lymph node lesions. Histopathology showed not only classic yeast-like fungi in proliferative macrophages, but also granulomas, gaseous necrosis, and small abscesses, suggesting that the immune system can play a defensive role.

There were relatively few cases of *Penicillium marneffei* infection among HIV-negative patients with and without underlying disease. The current recommendation for severe disease in HIV-positive patients is intravenous amphotericin B at a dosage of 0.6 mg∙kg^−1^∙d^−1^ for 2 weeks, followed by oral itraconazole 200 mg twice daily for 10 weeks. For mild disease, the recommendation is oral itraconazole at a dose of 200 mg twice daily for 8–12 weeks [6].

In this study, we found that HIV-negative patients with underlying disease had a worse prognosis and greater treatment difficulty than those without underlying disease. The reason for this is that HIV-negative patients with underlying disease have more obvious immune deficiency and CD4+ T cell-mediated immune deficiency than patients without underlying disease. Some diseases have the potential to damage immune function, especially CD4 T-cellular immune function. The majority of patients in this study received treatment for *Penicillium marneffei* infection with intravenous amphotericin B at a dosage of 0.6 mg∙kg^−1^∙d^−1^ for 4–8 weeks, followed by oral itraconazole or fluconazole 200 mg twice daily for 6–12 months, even treated for up to 2 years, which was significantly longer than the current recommendation for severe disease in HIV-positive patients. Oral itraconazole at a dosage of 200 mg once daily has been recognised as secondary prophylaxis. Thus, timely, effective, and longer courses of antifungal treatment, combined with support of the immune system, are important to improve patient prognosis. The longest duration for relapse was 4 years in this study; therefore, longer-term secondary prophylaxis and follow-up may be necessary to prevent relapse and improve prognosis. The important factors to assess when to discontinue secondary prophylaxis treatment include lymphocyte cell counts, CD4 cell counts, CD4 T-cell percentages, lung and bone imaging, and negative culture of clinical specimens.

## Conclusion

*Penicillium marneffei* can infect individuals without HIV infection and can cause fatal systemic mycosis. There were no clear differences in clinical manifestations among HIV-negative patients with and without underlying disease. However, *Penicillium marneffei* in patients without HIV infection, in combination with certain diseases, may cause immune function decline and a deficiency in T-cell-mediated immunity during the course of the disease. Underlying disease, CD4 cell percentage, and T lymphocyte cell percentage may be potential risk factors affecting prognosis. Timely, effective, and longer courses of antifungal treatments, in combination with treatments to improve immune function, are important in improving prognoses.

### Consent

Written informed consent was obtained from the patients for publication of this article and any accompanying images. Copies of the written consents are available for review.
